# RETRACTION: Kinesin Family Member 18A (KIF18A) Contributes to the Proliferation, Migration, and Invasion of Lung Adenocarcinoma Cells In Vitro and In Vivo

**DOI:** 10.1155/dim/9808595

**Published:** 2025-05-07

**Authors:** Disease Markers

RETRACTION: F.-T. Chen and F.-K. Zhong “Kinesin Family Member 18A (KIF18A) Contributes to the Proliferation, Migration, and Invasion of Lung Adenocarcinoma Cells In Vitro and In Vivo,” *Disease Markers*, no. 2019 (2019): 1–9. https://doi.org/10.1155/2019/6383685.

The above article, published online on 22 October 2019 in Wiley Online Library (wileyonlinelibrary.com), has been retracted by John Wiley & Sons Ltd.

The retraction has been agreed following an investigation of the concerns raised by *Hoya camphorifolia* on PubPeer [1], which identified multiple instances of figure duplication.

Specifically:

  - Figure 2b: The Western Blot bands for KIF18A and *β*-actin are identical to the bands corresponding to Ki67 and *β*-actin in Figure 2c of [2].
  - Figure 3c: The images of the wound closure assay have been reused (except the image on the bottom right) in at least 3 other publications (Figure 3c in [3], Figure 3c in [4], and Figure 3c in [5]).
  - Figure 3d: The “A549-Control” panel is identical to “U-2 OS-Control” panel in Figure 3d of [3], parts of which have also been spotted in Figure 3d of [5] and Figure 3b of [6].
  - Figure 4a: The tumor images appear identical to the tumors shown in Figure 6a of [7].
  - Figure 4b: The images of the 2 tumors have been duplicated in at least 2 other papers (Figure 5b in [8] and Figure 5b in [9]) and described as different tumor types.
  - Figure 4c: The immunohistochemistry image of KIF18A tumor tissue on the right has been duplicated in at least 2 other publications (Figure 6d (right) in [7], Figure 5c (right) in [8]), while the image on the left is a duplicate of Figure 5c (left) 9 in [10].

As a result of the investigation, the data and conclusions of this article are considered unreliable.

The authors were informed of the decision to retract the article but did not provide a response.
